# Innovative spectrofluorometric method for determination of harmaline and harmine in different matrices

**DOI:** 10.1038/s41598-023-46041-y

**Published:** 2023-11-15

**Authors:** Ahmed H. Abdelazim, Majed A. Algarni, Atiah H. Almalki

**Affiliations:** 1 Pharmaceutical Analytical Chemistry Department, Faculty of Pharmacy, Al-Azhar University, Cairo, 11751 Egypt; 2 Department of Clinical Pharmacy, College of Pharmacy, Taif University, P.O. Box 11099, 21944 Taif, Saudi Arabia; 3 Department of Pharmaceutical Chemistry, College of Pharmacy, Taif University, P.O. Box 11099, 21944 Taif, Saudi Arabia; 4 Addiction and Neuroscience Research Unit, Health Science Campus, Taif University, P.O. Box 11099, 21944 Taif, Saudi Arabia

**Keywords:** Chemistry, Analytical chemistry

## Abstract

Harmaline and harmine are naturally occurring closely related β-carboline alkaloids found in Peganum and Banisteriopsis plants. They have historical significance in traditional practices due to their potential psychoactive and therapeutic properties. Herein, a highly sensitive spectrofluorometric method was developed for the quantifying of harmaline and harmine in diverse matrices, including pure forms, seed samples, and spiked plasma. The procedures lie in addressing the challenge of overlapping fluorescence spectra exhibited by harmaline and harmine through the incorporation of hydroxypropyl-β-cyclodextrin, altering their chemical properties and fluorescence characteristics. Synchronous fluorescence measurements coupled with first derivative mathematical technique make it possible to distinguish between the harmaline and harmine at 419 and 456 nm, respectively. The method effectiveness is demonstrated through spectral analysis, optimization of the measurement conditions, adopting validation parameters and application to the pure form, seed samples and spiked human plasma. This methodology facilitates accurate determination of these alkaloids over the concentration range of 10─200 ng/mL. Thus, the developed approach provides a robust mean for the precise determination of harmaline and harmine, contributing to analytical chemistry's ongoing efforts to address complex challenges in quantification across diverse matrices.

## Introduction

**Figure 1:**
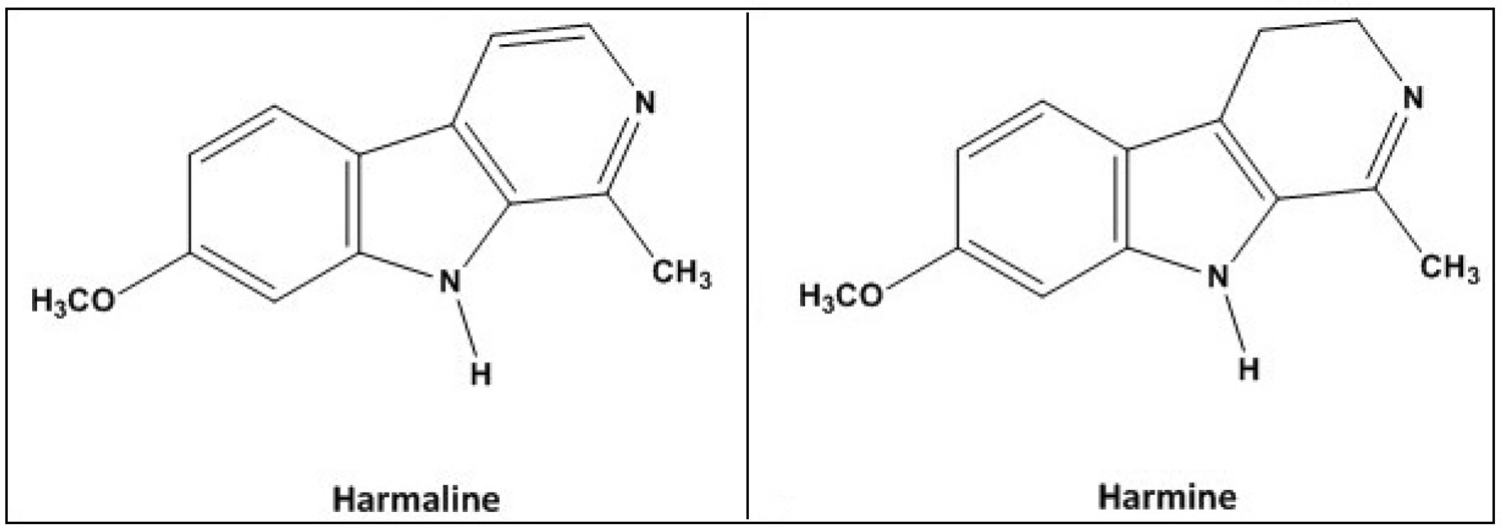
Structural formula of harmaline and harmine.

Harmaline (HL) and harmine (HM), Fig. 1, are natural β-carboline alkaloids found in Peganum and Banisteriopsis plants. They hold historical importance in traditional practices and modern research due to their suggested psychoactive and therapeutic effects. Used in ancient ceremonies for centuries, they induce altered consciousness and healing experiences. Investigated for pharmacological effects, including monoamine oxidase inhibition, they could treat depression and neurodegenerative disorders^1–3^. A range of edible plants, including wheat, rice, maize, soy, mushrooms, grapes, pineapple, bananas, and almonds, as well as fermented drinks like wine, beer, whisky, and brandy have also been shown to contain both HL and HM^4^. These compounds are generated during the cooking of meats and fish, and cigarette smoke have been recognized as significant sources of HL and HM, each showing varying quantification levels. Moreover, HL and HM have also been found in human tissues and various bodily fluids, underscoring their widespread occurrence^5,6^.

The field of analytical chemistry continually seeks innovative methodologies to address complex challenges encountered in the precise quantification of various compounds across diverse matrices. Therefore, development of a highly sensitive spectrofluorometric approach for the detection of HL and HM in a variety of matrices, such as pure forms, seed samples, and spiked human plasma, is the aim of this work. The key novelty of this research lies in developing innovative approach to address the challenge of overlapping fluorescence spectra exhibited by HL and HM. Through the incorporation of hydroxypropyl-β-cyclodextrin, that affects the chemical properties and subsequent fluorescence characteristics, and employing synchronous fluorescence measurements along with mathematical derivative techniques, Nano level quantification of HL and HM was achieved. This approach allows accurate determination in of HL and HM in pure forms and complex matrices; seed samples and spiked plasma.

## Experimental

### Materials and reagents

The reference standard, HL and HM, were obtained from Sigma-Aldrich, Germany. HPLC grade solvents including methanol, acetonitrile and chloroform were also acquired from Sigma-Aldrich, Germany. 2-Hydroxypropyl-β-cyclodextrin (HP-β-CD), also acquired from Sigma-Aldrich, Germany. A phosphate buffer solution with a pH of 7.5 was prepared following the U.S. Pharmacopeia guidelines, utilizing specified volumes of monosodium phosphate and disodium phosphate solutions. Peganum harmala seeds were obtained from the local market that complied with relevant guidelines. Healthy volunteers at the Al-Azhar University Hospital in Damietta, Egypt, who gave their informed consent before participating in the study were the source of the drug-free human plasma. The Committee on Research Ethics of Al-Azhar University in Egypt gave its approval to this work. In general, described procedures were carried out in accordance with relevant guidelines.

### Apparatus

Jasco FP-6200 Spectrofluorometer (Japan).

### Standard solutions

HL and HM stock solutions at a concentration of 100 μg/mL, was created by dissolving 10 mg of each respective reference standard in methanol (50 mL), which was then was then increased to a final volume of 100 mL with the same.

### Construction of calibration graphs

In a series of 10-mL volumetric flasks, diluted solutions of HL and HM in concentrations ranging from 0.1 to 2 g were made. Each flask received 2 mL of HP-β-CD and 2 mL of phosphate buffer solution with a pH of 7.5. To guarantee thorough dissolution and the development of inclusion complexes, the mixture was agitated for a full hour. The volume was then adjusted to the final level using methanol. These prepared solutions' synchronous fluorescence spectra were produced by scanning both monochromators at a fixed wavelength difference (Δλ) of 50 nm. The first derivative for each synchronous spectrum was collected with 9 data points in order to further analyse the spectra. For HL and HM, the peak amplitudes of the first derivative were determined to be at 419 nm and 456 nm, respectively. The results from the first derivative spectra were plotted against the final HL and HM concentrations in ng/mL to create calibration curves.

### Procedure for determination of the isolated HL and HM from the seed sample

Two grams of dried and powdered Peganum harmala seeds were subjected to extraction using 50 mL of methanol for a duration of 1h at room temperature. The resulting extracts were subsequently evaporated using a rotary evaporator. The resulting residue was then treated with 25 mL of hydrochloric acid (2% v/v) and subsequently filtered. The filtrate underwent a three-step extraction process with 10 mL portions of petroleum ether to eliminate impurities. The aqueous acidic layer was adjusted to a pH of 10 using ammonia and then subjected to a triple extraction with 25 mL portions of chloroform. The chloroform layer was evaporated to dryness, yielding a residue of total alkaloid with a mass of 0.17 g. The alkaloid residues were dissolved in 100 mL of methanol. Subsequently, the HL and HM were screened and quantified using the methodology outlined in the procedure for constructing calibration graphs.

### Procedure for determination of HL and HM in the spiked human plasma sample

The preparation of various human plasma samples spiked with various amounts of HL and HM was done. This was achieved by transferring measured portions of HL and HM at varying concentrations into a set of 10-mL centrifugation tubes. 0.1 mL of human plasma and 5 mL of acetonitrile were added to each tube. The tubes were stirred for one minute on a vortex mixer before being centrifuged for 30 min. The resultant supernatants were dried off by evaporation. Subsequently, the residues were reconstituted using an appropriate volume of methanol, along with 3 mL of phosphate buffer at pH 7.5, and then adjusted to the final volume with methanol in 10-mL volumetric flasks. A parallel blank sample was also prepared under identical conditions.

Subsequently, the samples underwent analysis following the procedure outlined in the calibration graph construction method.

### Ethics approval and consent to participate

This work was approved approved by the Committee of Research Ethics, Faculty of pharmacy, Al-Azhar University, Egypt.

## Results and discussion

Preliminary examination of the fluorescence spectra of HL and HM reveals that their spectra closely overlap, and the introduction of HP-β-CD induces changes in their respective fluorescence spectra. Moreover, employing synchronous scanning in conjunction with mathematical differentiation facilitates the selective quantification of HL and HM, effectively resolving the overlapping fluorescence spectra. The established approach successfully enables quantification of HL and HM in their pure forms, seed samples, and spiked human plasma samples.

### Spectral fluorescence characteristics

**Figure 2:**
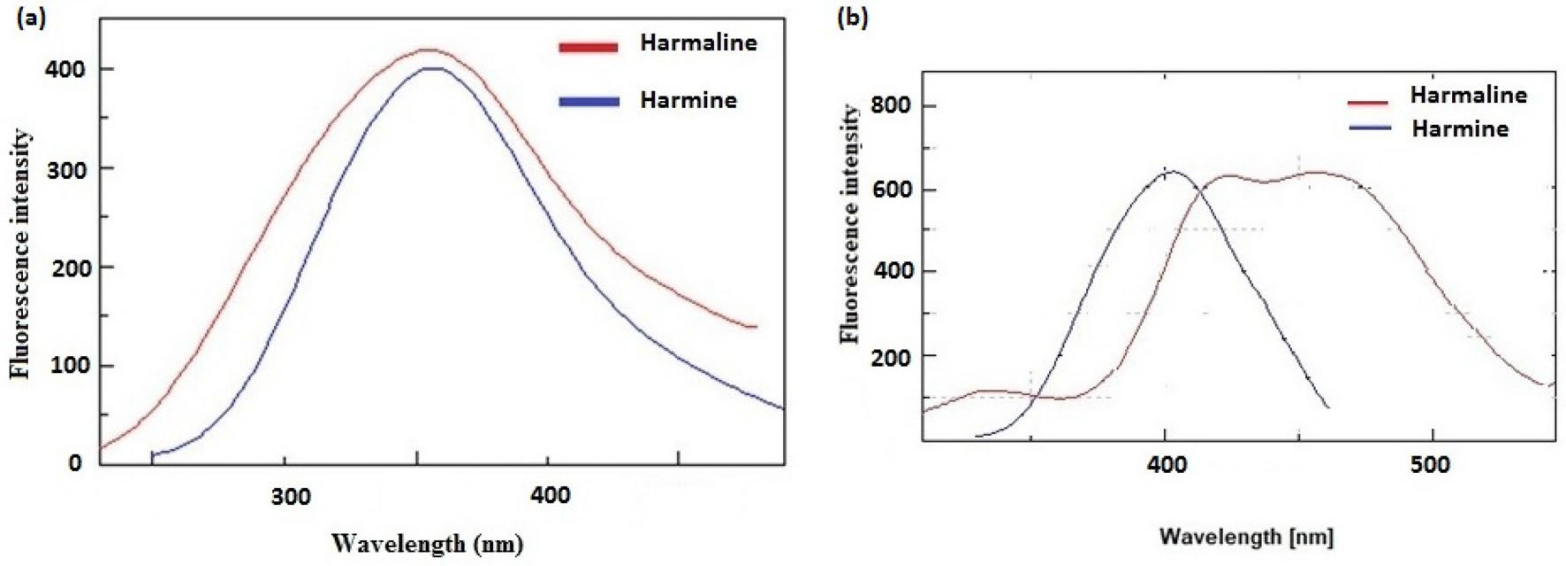
(**a**) Overlay emission spectra of harmaline and harmine, (**b**) Overlay emission spectra of harmaline and harmine after incorporation of hydroxypropyl-β-cyclodextrin.

HL and HM are closely related β-carboline alkaloids with distinctive secondary heterocyclic amines and an indole skeleton-fused pyridine ring^7,8^. These alkaloids display distinct fluorescence properties and unique acid–base behaviors in both their ground and excited states. These features can be attributed to the presence of pyridine nitrogen, acting as a base and susceptible to protonation, as well as the presence of pyrrolic nitrogen, acting as an acid and releasing a proton within a basic environment. Upon excitation at 285 nm, HL and HM exhibit strongly overlapping fluorescence emission spectra in the range of 300 nm to 400 nm, as depicted in Fig. 2. This overlapping characteristic poses a challenge for the determination of HL and HM.

The incorporation of HL and HM with HP-β-CD alters their chemical characteristics and leads to changes in the acid–base traits of the resulting complexes, consequently influencing their fluorescence emission properties.

The hydrophobic cavity within HP-β-CD can provide significant modification of the physicochemical properties of compounds upon inclusion and complex formation. This can be attributed to the influence of electron donor substituents or the surrounding environment on the stabilization of the corresponding complex. The complexation with HP-β-CD has the capacity to induce alterations in the pKa values of the involved HL and HM. Moreover, HP-β-CD exerts a discernible impact on the apparent ionization constants of both HL and HM, subsequently affecting the association constants of these compounds. This is further exemplified by the changes in binding constants observed when alkaloids are included within the HP-β-CD structure. The excited states of HL and HM are protected within the cavity of HP-CD, enabling proton transfer^9^. This phenomenon results in shifts within the fluorescence emission spectra of HL and HM, though a certain degree of overlap remains apparent. Specifically, following excitation at 285 nm, the maximum fluorescence emissions occur at 400 nm and 455 nm for HL and HM, respectively as illustrated in Fig. 2.

**Figure 3:**
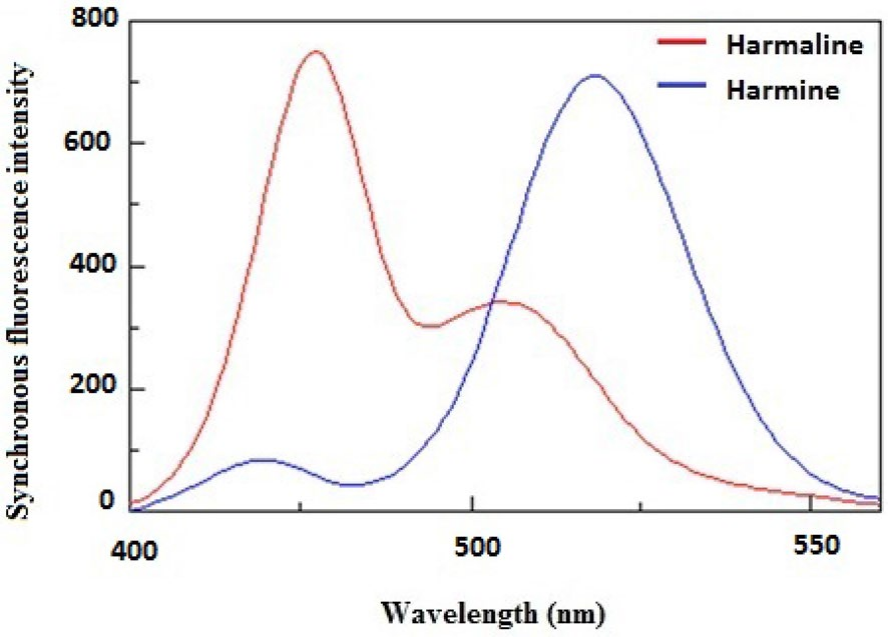
Synchronous fluorescence spectra of the harmaline and harmine after incorporation of hydroxypropyl-β-cyclodextrin.

**Figure 4:**
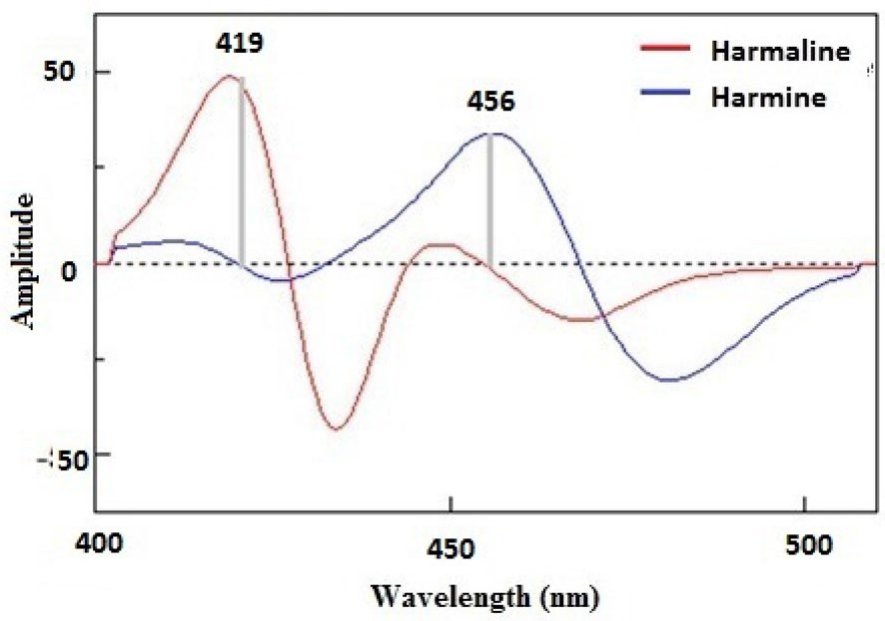
First derivative of the synchronous fluorescence spectra of harmaline and harmine after incorporation of hydroxypropyl-β-cyclodextrin.

This issue of overlapping was successfully resolved through the utilization of synchronous fluorescence measurements^10–14^, which aided in the development of distinct and narrow spectra. The synchronous fluorescence of both HL and HM was recorded at Δλ = 50 nm, as illustrated in Fig. 3, followed by the application of the first derivative mathematical technique. The resultant first derivative values of the synchronous spectra for HL and HM were effectively separated, enabling the determination of HL at 419 nm and HM at 456 nm without encountering any interference, as depicted in Fig. 4.

### Optimization of the measurement conditions

#### Selection of optimum Δλ

Synchronous fluorescence performance is influenced by selecting the optimum scanning Δλ in requisites of sensitivity, resolution, and quality. The scanning of synchronous fluorescence spectra is influenced by the chosen Δλ value. To explore this, Δλ values in the range of 20–80 nm were examined. Among these, the most optimal value was determined to be Δλ = 50 due to its ability to provide both high resolution and sharply defined peak shapes.

#### Effect of diluting solvent

**Figure 5:**
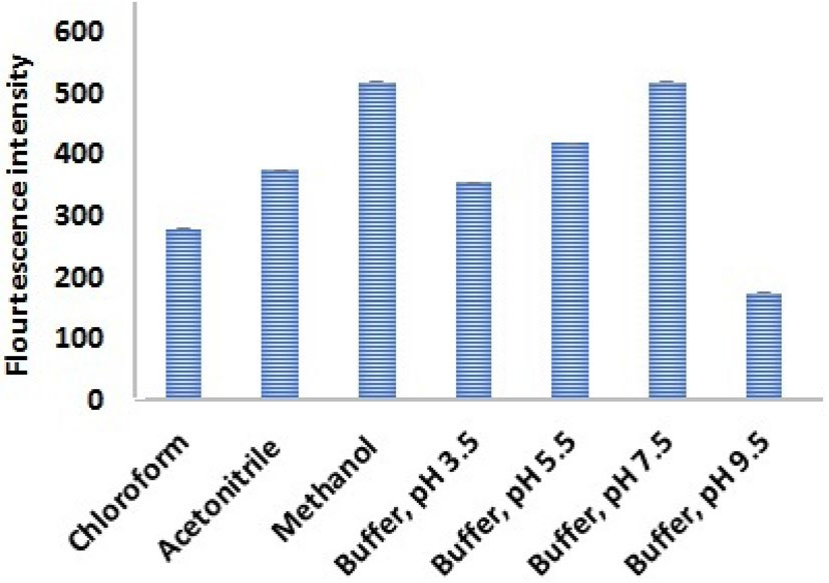
Study of solvents and buffer at different pH values toward selecting the best conditions.

Different solvents, namely methanol, acetonitrile and chloroform on the intensity of the synchronous fluorescence spectra was investigated. Among these, methanol proved to be more suitable for the quantitative analysis of HL and HM in their binary mixtures, as shown in Fig. 5.

#### pH and buffer conditions study

A comprehensive exploration involving different buffers, spanning a wide pH range, was conducted to identify the optimal pH conditions for the precise determination of HL and HM. The findings indicated that phosphate buffer at pH 7.5, 2 mL was particularly suitable as illustrated in Fig. 5.

### Method validation

Validation was carried out in accordance with ICH recommendations^15^. The calibration plots exhibited linear relationship over the concentration range of 10–200 ng/mL for both HL and HM, with regression parameters provided in Table 1. Residual standard deviation (SD) and slope were used to estimate LOD and LOQ, higher quantification and detection limits were obtained as indicated in Table 1. The method's robustness was established through minor variations in conditions, affirming its stability and reliability, as detailed in Table 1. By examining three duplicates of HL and HM samples at concentrations of 10, 50, and 100 ng/mL, accuracy and precision were assessed. These analyses were conducted on the same day and then carried out three more times. Accuracy was verified through mean percent recovery (%R) calculations, while precision was assessed by percent relative standard deviation (%RSD), the findings showed that the method met the requirements for both accuracy and precision, as presented in Table 1. Furthermore, by examining combinations with varying HL and HM concentrations in synthetic produced mixtures, the method's specificity was evaluated. The results, shown in Table 2, confirmed the procedure's specificity and its reliability for selective analyte determination.

**Table 1:** Regression and validation data for the determination of harmaline and harmine using the proposed method.

Parameters	Harmaline	Harmine
Wavelength (nm)	419	456
Linearity range (ng/mL)	10–200	10–200
LOD (ng/mL)	2.51	1.82
LOQ (ng/mL)	7.53	5.46
Slope	0.011	0.024
Intercept	0.762	0.750
Coefficient of determination (r^2^)	0.9998	0.9997
Accuracy (%R)^a^	98.20	99.65
Repeatability Precision (RSD)^b^	0.805	0.878
Intermediate precision (RSD)^b^	0.780	0.799
Robustness (%R ± RSD)		
Buffer pH (± 0.5)	100.33 ± 0.804	98.55 ± 0.651
Buffer volume (± 0.25 mL)	98.26 ± 0.708	99.20 ± 0.840
HP-β-CD volume (± 0.1 mL)	98.54 ± 0.698	100.24 ± 0.925

^a^Average of nine determinations (three concentrations repeated three times).

^b^RSD of nine determinations (three concentrations repeated three times).

**Table 2:** Application of the proposed method for the determination of harmaline and harmine in synthetic prepared mixtures.

Harmaline		Harmine	
Added, ng/mL	Found, ng/mL (%R)	Added, ng/mL	Found, ng/mL (%R)
75	74.29 (99.05)	25	24.95 (99.80)
100	98.24 (98.24)	100	99.62 (99.62)
25	24.85 (99.40)	75	74.57 (99.42)
Mean ± RSD	98.89 ± 0.98	Mean ± RSD	99.61 ± 0.425

### Application for the seed sample extract

The extraction of HL and HM from dried and powdered Peganum harmala seeds was achieved. Two grams of the seeds were extracted, concentrated and yielded a dry residue of a total alkaloid mass of 0.17 g. The analysis of solution extracts containing both HL and HM, was executed in accordance with the method described earlier. Notably, a distinct disparity in concentration emerged between HM and HL within the sample extract. Specifically, the concentration of HM exhibited a notable elevation, registering at 46.35 ng/mL, while the HL concentration was comparatively lower at 33.18 ng/mL.

### Application for spiked human plasma samples

The reliability of the established methodology in quantifying HL and HM concentrations within spiked human plasma was thoroughly examined. Demonstrating an exceptional sensitivity for detection, the method facilitated precise quantification of both HM and HL. With a lower C_max_ of 67.05 ng/mL for HM and 117.80 ng/mL for HL^16^, the developed approach emerged as a remarkably sensitive analytical instrument for assessing plasma samples. The method's effectiveness in determining the spiked samples was clearly demonstrated by the recovery values consistently falling within the acceptable range, as delineated in Table 3.

**Table 3:** Application of the proposed method for the determination of harmine and harmine in spiked plasma samples.

Harmaline		Harmine	
Added, ng/mL	Found, ng/mL (%R)	Added, ng/mL	Found, ng/mL (%R)
67	65.71 (98.07)	200	197.08 (98.54)
120	118.59 (98.82)	120	117.55 (97.95)
200	196.08 (98.04)	67	65.99 (98.49)
Mean ± RSD	98.31 ± 0.799	Mean ± RSD	98.32 ± 0.952

## Conclusion

An innovative spectrofluorometric method was developed for the determination of harmaline and harmine in various complex matrices, including pure forms, seed samples and spiked human plasma. By incorporating hydroxypropyl-β-cyclodextrin to modify the chemical properties of harmaline and harmine and employing synchronous fluorescence measurements along with mathematical derivative techniques, the challenge of overlapping fluorescence spectra was effectively addressed. The method's validation, encompassing accuracy, precision, limits of detection, and robustness, attested to its reliability and applicability. The method provides successful determination of these alkaloids in different matrices and introduces applied procedures for various applications, including pharmacological and toxicological studies. Moreover, the proposed approach showcases the continual advancements in analytical methodologies to tackle complex analytical challenges.

## Data Availability

The datasets used during the current study are available from the corresponding author on reasonable request.
